# Infection of the Nose in Diphtheria

**Published:** 1907-07-27

**Authors:** 


### Infection of the Nose in Diphtheria.

Ever since diphtheria has been recognised as a specific disorder, the occurrence of coryza, membranous or otherwise, in the course of the disease has been a subject of interest and a cause of controversy. It is of importance both in the causation and the prognosis, and more so than in either of these, in the prevention of diphtheria. Pierre Bretonneau, of Tours, who was the first to realise that various forms of "malignant sore throat" were in reality one and the same disease, noticed that a nasal discharge frequently preceded a severe attack of diphtheria, and believed that the disease always had its origin in the nose, and that the membrane passed thence to the fauces. Trousseau went to the opposite extreme and denied that diphtheria ever originated in the nose. Since the discovery of the Klebs-Loeffler bacillus, it has been found that this organism is frequently present in the nose in cases of pharyngeal diphtheria, whether hypersecretion and membrane be present or not. Of the cases in which a definite false membrane is present in the nose, there appear to be two distinct varieties, similar in their local features, but sharply contrasted in their general effects. In true nasal diphtheria the affection may be primary in the nose, though the fauces are usually also involved, and the disease is clinically a severe type of diphtheria; on the other hand, in membranous or fibrinous rhinitis the membrane is generally limited to the nose, and constitutional symptoms are slight.

The question of diphtheritic infection by the nose has recently been revived by Sevestre who, in 1904, stated that a history of nasal discharge could often be elicited in cases of children suffering from faucial diphtheria, and Marfan, in 1905, believed the pharyngeal tonsil to be the site of the original infection. Dr. J. D. Rolleston, in a recent paper based on 1,200 consecutive cases, discusses the subject of nasal discharge in diphtheria and its bearing on the severity of the disease. He found that 26.91 per cent. of cases had a discharge from the nose either on admission or developed subsequently during the acute stage; there was a history of recent nasal discharge which had ceased before admission in a further 14.75 per cent., making a total of 41.6 per cent. Of the 177 cases who had had a nasal discharge which ceased before admission only four had received antitoxin, and two of these had been injected on the day of admission; therefore, it is clear that the discharge may cease spontaneously. It was also found that this "self-limiting" discharge was most frequent among the milder cases and became progressively rarer among those suffering from severer forms of the disease; on the other hand, cases with persistent nasal discharge, or those in whom the discharge developed during the acute stage after admission, were usually severe, and were found with diminishing frequency among the milder cases. This distinction seems to be borne out also in the incidence of albuminuria and paralysis, and in the percentage of fatal cases; thus there appear to be two distinct classes of case with nasal discharge. In the one, the rhinorrhœa is a very early symptom, disappears quickly, and is associated with a mild type of the disease; in the other, the discharge persists or developes later during the acute stage, and is accompanied by a higher mortality and a greater incidence of complications.

It is not stated how frequently the discharge contains diphtheria bacilli in a condition of virulence, nor how long the rhinorrhœa may persist during convalescence. The patient is generally considered to be a source of infection as long as the throat contains the Klebs-Loeffler bacillus, but there is a strong possibility that the nose may continue to harbour these organisms after they have disappeared from the throat and, in view of the persistency of the diphtheria bacillus, the nasal discharge in diphtheria may prove to be an effective agent for the spread of infection, and its frequency thus becomes of great importance.

